# Factors Associated with Tuberculosis Outcome in a Hyperendemic City in the North of Brazil

**DOI:** 10.3390/healthcare11040508

**Published:** 2023-02-09

**Authors:** Gabriel Fazzi Costa, Juliana Conceição Dias Garcez, Weber Marcos, Ana Lúcia da Silva Ferreira, Jorge Alberto Azevedo Andrade, Yan Corrêa Rodrigues, Luana Nepomuceno Gondim Costa Lima, Emilyn Costa Conceição, Karla Valéria Batista Lima

**Affiliations:** 1 Program in Parasitic Biology in the Amazon Region (PPGBPA), State University of Pará (UEPA), Belém 66087-662, PA, Brazil; 2 Department of Health of Pará State, EpiSUS Intermediário, Belém 66093-677, PA, Brazil; 3 Program in Epidemiology and Health Surveillance (PPGEVS), Evandro Chagas Institute (IEC), Ananindeua 67030-000, PA, Brazil; 4 Bacteriology and Mycology Section, Evandro Chagas Institute (SABMI/IEC), Ministry of Health of Brazil, Ananindeua 67030-000, PA, Brazil; 5 Division of Molecular Biology and Human Genetics, Faculty of Medicine and Health Sciences, Stellenbosch University, Stellenbosch 7602, South Africa

**Keywords:** tuberculosis, outcome, epidemiology, Brazil

## Abstract

Ananindeua city, State of Pará, North of Brazil, is a hyperendemic area for tuberculosis (TB), with a cure rate below the recommendation by the Brazilian Ministry of Health. We aimed to describe: (I) the TB incidence coefficient of Ananindeua municipality comparatively against Brazilian data; (II) TB treatment outcomes; (III) to compare the socioeconomic and epidemiological characteristics of abandonment versus cure outcome; and (IV) to evaluate the risk factors associated with TB treatment abandonment in Ananindeua city, from 2017 to 2021. This is a retrospective, descriptive, and cross-sectional epidemiological study which used secondary TB entries. Data were analyzed by linear regression, descriptive statistics, and associations were made using the Chi-square test and G-test, followed by univariate and multivariate logistic regression analyses. Cure rates ranged from 28.7% to 70.1%, abandonment between 7.3% and 11.8%, deaths from the disease ranged from 0% to 1.6%, and drug-resistant tuberculosis (TB-DR) rates had frequencies from 0% to 0.9%. Patient transfer rates to other municipalities were between 4.9% and 12.5%. The multivariate analysis showed that alcohol is almost 2 times more likely to lead an individual to abandon treatment and use of illicit drugs was almost 3 times more likely. Individuals between 20 and 59 years of age were also more likely to abandon treatment almost twice as often. Finally, data obtained in the present report is of great relevance to strengthen epidemiological surveillance and minimize possible discrepancies between the information systems and the reality of public health in high endemicity areas.

## 1. Introduction

Tuberculosis (TB) is a curable disease in virtually all cases in individuals who have bacilli that are sensitive to anti-tuberculosis (anti-TB) drugs, if treatment is correctly instituted and maintained to eliminate the disease-causing agent [1]. Indeed, TB treatment interruption entails a series of problems for individuals and society, such as death, sequelae, outbreaks and antibiotic-resistant strains emergence, limitations for drug therapy, increase of health systems costs, and permanence of dissemination foci within community environments [2].

In Brazil, TB treatment adherence is associated with several biological and social factors, which may be closely linked to the patient themself, such as health, psychological, cognitive, behavioral, and belief issues; to the disease, such as condition severity; and to the treatment, including access to health services. Despite several advances in TB treatment, adult male individuals from low socioeconomic conditions, black-skinned people, as well as institutionalized patients are at higher risk of the disease [1,3,4,5].

In this context, it is estimated that the TB abandonment rate in Brazil is around 9% of cases, ranging from 2.8% to 15.9% among the states of the federation [6]. In the Northern Brazilian State of Pará, between 2016 and 2019, an average of 11.7% of individuals comprised the treatment abandonment rate [7]. These numbers are above the limit of 5% established as a parameter for this indicator by national and international health agencies [1,6,8,9,10].

Also, in 2021, the city of Ananindeua, State of Pará, had an incidence of 73.8 cases of tuberculosis per 100,000 inhabitants, an average higher than the state and national averages, which were 46.9% and 32.0% per 100,000 inhabitants, respectively [7]. A recent epidemiological study with data from 2018–2020 demonstrated that Ananindeua is a hyperendemic area for TB, harboring a cure rate of 60.31%, which was below the recommendation by the Brazilian Ministry of Health. The quality indicators showed many weaknesses (non-availability of smear microscopy, culture, and rapid molecular test (RMT) tests; and, among others, failure to fill in essential variables for TB surveillance [11]).

Data from the Brazilian Notifiable Diseases Information System (SINAN) has shown that TB incidence in Ananindeua municipality between 2017 and 2021 was between 61.3% and 93.9% per 100,000 inhabitants, and the treatment abandonment rate was between 7.3% and 11.8% of cases closed [12]. Based on that, we aimed to (i) describe the TB incidence coefficient of Ananindeua municipality comparatively against Brazilian data; (ii) to describe the TB treatment outcomes; (iii) to compare the socioeconomic and epidemiological characteristics of abandonment versus cure outcomes; and (iv) to evaluate the risk factors associated to the TB treatment abandonment in Ananindeua municipality from 2017 to 2021.

## 2. Materials and Methods

### 2.1. Study Design

This is a retrospective, descriptive, and cross-sectional epidemiological study which used secondary TB entries from the municipality of Ananindeua, State of Pará, Brazil, between January 2017 to December 2021. We followed the guidelines for reporting observational study recommendations based on the Strengthening the Reporting of Observational Studies in Epidemiology (STROBE) Statement [13,14].

### 2.2. Study Scenario

The present study was conducted with data from the city of Ananindeua/PA and data provided by the Health Secretariat of the State of Pará (SESPA). This data is sent by each municipality/city to the state secretariats and, after consolidation, are sent to the Ministry of Health, and added to the National System of Diseases and Notifications (SINAN) of the Department of Informatics of the Unified Health System (DATASUS) of the Ministry of Health of Brazil. Ananindeua has an estimated population of 540,410 inhabitants with a territorial extension of 190,451 km, located in the 6th microregion of the state of Pará, bordering the municipality of Belém, the state capital, and the city of Marituba [15,16].

### 2.3. Selection Criteria and Data Collection

The data obtained refer to cases reported in the municipality between 2017 and 2021. The outcomes included in the statistical analyzes were treatment abandonment and cure. The other types of outcomes were only described. The total sample of entries with a closure status due to cure and abandonment was 1599 cases. However, some of these entries had no response for some of the variables of interest in the study, and therefore were not considered in the logistic regression analysis. The total number of entries with all fields of interest filled in was 1345 (used in the univariate and multivariate analyses). The data were collected in May 2022. The variables of interest analyzed were sex, age, race, education, participation in a social cash transfer program, type of entry, form, Directly Observed Treatment (DOT), alcoholism, smoking, use of illicit drugs, and HIV coinfection.

The definition of cure is when the patient presents two negative smears, one in any month of follow-up and another at the end of the treatment (5th or 6th month), for longer treatments the last two months are considered. Patients who completed the treatment with no evidence of failure and were discharged based on clinical and radiological criteria, due to the impossibility of performing bacilloscopy exams, were also discharged for cure. Abandonment, on the other hand, is defined as the use of medication for 30 days or more, followed by discontinuation for 30 consecutive days or more [1].

### 2.4. Statistics Analysis

The incidence coefficient is the parameter that allows comparing the number of new cases of a given disease between cities, states, and countries, as it estimates the number of people affected by a disease per 100,000 inhabitants.

The TB incidence coefficients were calculated using the population projections of the Statistical Yearbook of Pará 2021, the Projection of the population of Brazil released by the Fundação Amazônia de Amparo a Estudos e Pesquisas (FAPESPA) [15], and the Brazilian Institute of Geography and Statistics (IBGE) [16]. Linear regression analysis was performed to determine the tendency for growth or decrease in incidence between the years analyzed, with a significance level of *p* < 0.05.

Treatment abandonment and cure data were analyzed and are presented according to the absolute and relative frequency and associations were analyzed using the Chi-square test and G-test, with a significance level of *p* < 0.05. Variables that showed statistical significance were submitted for univariate and multivariate logistic regression analysis to identify risk factors associated with treatment dropout. For the uni- and multivariate regression analyses, only the 1345 entries that contained all the variables were considered. Some variables were categorized and dichotomized. All variables submitted for univariate logistic regression analysis with a *p*-value < 0.1 were considered eligible for multivariate analysis. The variable year of entry was included in the regression, using the year 2017 as a reference. Statistical tests were run using Bioestat software version 5.4 and Mnitab.

### 2.5. Ethics

Despite being a secondary data analysis database, with no direct contact with the researched individuals, the database contains personal information and therefore requires compliance with the ethical guidelines, established by law, and approved by the Research Ethics Committee of Evandro Chagas Research Institute, under standards 4.172.679 and CAEE 20821119.4.0000.0019.

## 3. Results

### 3.1. Number of New Reported Cases of Tuberculosis and Incidence Coefficient in Ananindeua, State of Pará and Brazil

The incidence of tuberculosis in Ananindeua has shown an increase, especially in 2019, which had the highest number of cases among the years studied. In 2021 there was a decrease in cases compared to the previous year, however, the number remains higher than in 2017 and 2018. The same is observed among the numbers of new cases reported in the state of Pará and in Brazil, with a high number of cases in 2019, followed by a slight decrease in the following years (Table 1).

**Figure 1:**
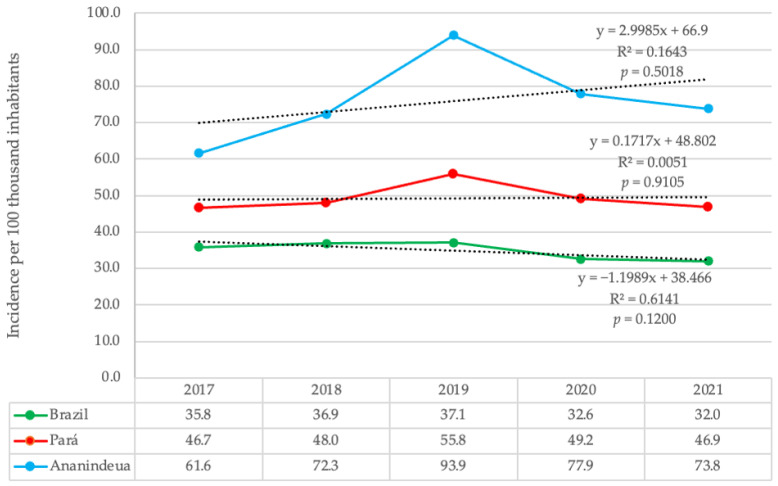
Trend of TB incidence coefficient in the Municipality of Ananindeua, state of Pará and Brazil from 2017 to 2021. Trend line represented by the dashed line.

The TB incidence in Ananindeua demonstrated rates higher than the state and national incidences throughout the period of this study, with a tendency for the trend to increase. The linear regression model adopted points to an increase of 2.99 cases per year per 100,000 inhabitants, however the coefficient of determination of the regression function shows that this trend was not representative (R^2^ = 0.1643), and the value of the test was not significant (*p* > 0.05). The TB incidence rate in Pará maintained a slight increase compared to that of Brazil, which showed a decrease. Both Ananindeua and the state of Pará and Brazil had the highest TB incidence rates in 2019 compared to other years, due to the increase in the number of cases for this year, as shown (Table 1, Figure 1).

### 3.2. Tuberculosis Treatment Outcomes Reported in Ananindeua

Between the years 2017 and 2021, 2339 entries were found, of which 431 (18.4%) did not have the closure status field filled in. Of this number, 1599 entries were selected, of which 1369 (58.5%) were of people who were discharged from TB treatment having been cured and 230 (9.8%) of people who abandoned treatment. These are the two variables that make up the comparison groups of the study. The remaining cases refer to the other scenarios for termination, as described in Table 2.

Cure rates were between 28.7% and 70.1%, abandonment rates were between 7.3% and 11.8%, deaths from the disease ranged from 0% to 1.6% of cases, and rates of drug-induced tuberculosis resistance (DR-TB) presented frequencies from 0% to 0.9%, between the analyzed years. Transfer rates of patients to other municipalities were between 4.9% and 12.5%.

### 3.3. Socioeconomic Characteristics of Abandonment Cases and Cure Outcome

Cases reported as treatment abandonment or cure of the disease, the outcomes of interest in the study, totaled 1599 entries between 2017 and 2021. In the comparison between the two groups (abandonment and cure), there was a significant difference for gender and age group. Males showed the highest frequency, with 154 (67%) among those who abandoned treatment (*p* = 0.0395). The adult age group, 20 to 59 years old, was also more present among dropouts, with 194 (84.3%, *p* = 0.0062).

The predominant race in both groups was brown/pardo, with 174 (75.7%) of those who abandoned treatment and 1055 (77.1%) of those who were cured. As for education, individuals who had incomplete elementary school education (*n* = 95, 41.3%) were more associated with the abandonment outcome, while complete elementary school education (*n* = 44, 19.1%) and complete high school education (*n* = 339, 24.8%) were more associated with individuals who achieved a cure (*p* < 0.0001). The schooling variable was one of the most unfilled fields in the entries, with a percentage of 23.1% of cases without information; for this reason, we chose to exclude this variable from the logistic regression analyses (Table 3).

### 3.4. Epidemiological Characteristics of Abandonment Cases and Cure Outcome

As for the type of admission, a new case related to the cure group with 1203 (87.9%, *p* < 0.0001) individuals and re-entry after abandonment was statistically significant among the abandonment group 186 (80.9%, *p* < 0.0001). The disease form showed no significant difference between the groups (*p* > 0.05). Directly Observed Treatment (DOT) also showed no significant difference between the groups and was not performed in most cases (N = 142, 61.7%).

Alcoholism, smoking, and the use of illicit drugs were associated with cases of abandonment, respectively. HIV negative serology was significant for the cure group with 630 cases (46.0%, *p* < 0.0001). It is noteworthy that in 47.3% of cases, almost half of the total sample, HIV serology was not performed, making it impossible to measure. For this reason, it was also decided to exclude it from the logistic rlegression analysis, since removing the entries without this information would substantially compromise the size of the study population (*n* = 757) (Table 4).

### 3.5. Risk Factors Associated with Treatment Abandonment

For the logistic regression analysis, only the entries that had all the variables filled out and that were significant in the Chi-square or G-test were considered. Of the 1599 entries, only 1345 were filled out. We chose to include the variable year, taking the year 2017 as a reference.

The results of the univariate analysis showed that age group (OR: 1.57; 95% CI: 1.04–2.38; *p*: 0.033), type of entry (OR: 0.49; 95% CI: 0.31–0.78; *p*: 0.002), alcoholism (OR: 2.72; 95% CI: 1.68–4.40; *p* < 0.001), smoking (OR: 3.33; 95% CI: 1.73–6.41; *p* < 0.001), drug use (OR: 4.23; 95% CI: 2.35–7.60; *p* < 0.001), and the year 2021 (OR: 1.47; 95% CI: 1.03–2.09; *p*: 0.032) were significantly associated with treatment dropout. In addition, multivariate analysis showed that the age group (OR: 1.55; 95% CI: 1.03–2.32; *p*: 0.035), alcoholism (OR: 1.74; 95% CI: 1.11–2.72; *p*: 0.016), illicit drug use (OR: 2.64; 95% CI 1.56–4.46; *p* < 0.001), and the year 2021 (OR: 1.45; 95% CI: 1.01–2.08; *p*: 0.043) are predictors associated with treatment abandonment (Table 5).

## 4. Discussion

The city of Ananindeua has higher incidence rates than the state of Pará and Brazil and is therefore considered a hyperendemic region for TB [11]. The incidence of cases in the municipality of Ananindeua has followed the state and national trends that saw an increase in cases from 2017 to 2019 and a discrete decrease in the years 2020 and 2021. The epidemiological scenario brought on by the COVID-19 pandemic and the measures to contain virus transmission influenced the detection of new cases, causing probable underreporting, and interfered with the continuity of ongoing treatments [17,18].

The study shows that in all the years analyzed, the cure rates for TB each year remained below 85%, the minimum recommended by the Brazilian Ministry of Health. The World Health Organization (WHO) recommends at least a 90% cure of identified cases [8,9]. Dropout rates were above 5%, the maximum recommended by the Ministry of Health in Brazil [1,10].

The municipalities of the state of Pará along with municipalities from other states were grouped in a cluster formed by 163 municipalities at high risk for treatment abandonment in the years 2012 to 2018, in a study on the spatial-temporal distribution of TB treatment abandonment in Brazil. In these municipalities, the average proportion of abandonment was 16.85% [6].

Although 2021 had only a 28.1% cure rate, we emphasize that this number may represent the incompleteness of the data due to the delay in updating the information passed on to the surveillance system. The information is passed on by each notifying unit to the municipal departments and later to the state departments until it reaches the Ministry of Health, where the consolidated information is published and becomes public domain. This data is updated as new information is passed on.

Surveillance data from the Tuberculosis Special Treatments Information System (SITE-TB) that make up another database for monitoring drug-resistant tuberculosis (DR-TB) cases, shows a difference between the numbers of DR-TB presented. According to the system, 12 cases of DR-TB were reported in 2017, 14 cases in 2018, 8 cases in 2019, 5 cases in 2020, and 10 cases in 2021 [19].

The SITE-TB is a complementary system to the Information System for Notifiable Diseases (SINAN), and the main tool for surveillance and management of TB cases with indication of special schemes. This inconsistency between data shows a communication gap between the systems since every confirmed case of TB should be notified in SINAN, regardless of the type of treatment to be administered [20].

The cases of transfer of patients to other municipalities totaled an average of 8.5% between the years analyzed. According to the National Plan for the End of Tuberculosis as a Public Health Problem in Brazil, this indicator, together with the cases in which the outcome of the treatment was not informed, compromises the actions to combat the disease. Since there is no information, the analysis of cure and abandonment indicators are impaired [10].

Other important variables also neglected were education, which presented a total of 23.1% of the total cases of cure and abandonment and was not filled out information, and the performance of HIV serology throughout treatment, which was not done in almost half (47.3%) of the cases of cure and abandonment, going against the advice of the Brazilian Ministry of Health’s recommendation to offer HIV testing to all TB cases [1]. TB/HIV coinfection is already well documented in the literature, with people living with HIV who are not on treatment having a higher risk for developing the disease, including its extrapulmonary form, and a higher risk of death, if bought to people not living with the virus [21,22,23].

Regarding socioeconomic characteristics, our results show an association between males and the group with the abandonment outcome, however, in the regression analysis, there was no significant difference. This may be due to factors such as alcoholism and the use of illicit drugs, which in our study were shown to be risk factors for abandonment, and are behaviors more observed among males [24].

Different studies point to an association between treatment abandonment and certain factors. Sociodemographic factors, such as the use of licit or illicit drugs, age, sex, education, income, housing, access to information and health services and stigmas, clinical factors such as co-infection with the HIV, history of abandonment, extrapulmonary TB and comorbidities, and treatment-related factors, such as the stage of treatment (usually at the beginning), adverse drug effects, and improvement in disease symptoms are some of the commonly reported factors [3,25,26,27].

Our results show that individuals considered to be alcoholics are almost 2 times more likely to abandon treatment, while those who use illicit drugs are almost 3 times more likely. In Belém/PA, a survey of patients undergoing retreatment for the disease observed that the use of illicit drugs and alcoholic beverages were among the most cited reasons why patients decided to discontinue treatment [27].

In the state of Ceará, it was observed that alcoholism increased the chances of abandoning treatment by 50% [5]. Another study carried out, this time in the capital of Ceará, also showed that in addition to alcoholism, the use of illicit drugs significantly increased the chances of abandonment [3].

In a systematic review, alcohol use was associated with a 1.5- to 2-fold increase in the odds of poor treatment outcomes in drug-sensitive TB and DR-TB, relative to minimal or no alcohol exposure. Alcohol and TB treatment were associated with higher odds of poor treatment outcomes, i.e., death, treatment failure, and loss to follow-up, in studies of drug-sensitive (OR 1.99, 95% CI 1.57–2.51) and multidrug-resistant TB patients (OR 2.00, 95% CI 1.73–2.32) [28].

The age group which presented with an increased risk of treatment abandonment, with 1.5-fold higher chance were those aged between 20 and 59 years. Our criteria for determining this age group were based on the population considered economically active. These results are concordant with another study that identified a higher risk of abandonment among younger adults aged between 20 and 40 years [3].

As for the analysis of the year, the year 2021 presented odds of 1.45. We consider that this finding should be analyzed more carefully over time, since the data for the year 2021 were not yet complete and many entries were not yet closed, due to the delay between the closure of the case and the arrival of information to the surveillance agencies. It is worth remembering that the minimum time for the treatment of tuberculosis is six months, at best, while the time for the individual to be considered in the treatment give-up group is 30 days after the date of the last dispensation of the drug.

For this reason, there may be a difference in the proportion of cases of cure and treatment abandonment that does not reflect the totality of cases in the year, if we analyze each year separately, so a data analysis is necessary when this period has the most entries with status closed.

In this study, the type of entry and smoking was associated with treatment abandonment in the univariate regression analysis, but it was not significant in the multivariate analysis. Factors such as participation in a cash transfer program, form of the disease and DOT were not statistically significant. While ethnicity, the type of entry and smoking were associated in the Chi-square tests, the G-test or univariate regression analysis, however, did not show significance in the final regression model. This may be because many exposure factors are related to each other but are not always the direct cause of a problem.

Low education commonly associated with cases of tuberculosis, was not included in the logistic regression analyses because many entries did not inform this data. However, low education is related to alcohol abuse [29]. The absence of many responses to the HIV serology status also resulted in the non-inclusion of this variable in the regression model. This lack of information can compromise the findings and generate results which differ from reality. For this reason, it is important from the point of view to strengthen surveillance systems so that the information is properly filled out, because it is from these findings that health policies are formulated [30].

The problem of incomplete information in the entries was identified in research on the evaluation of TB surveillance data in Brazil and points to the need for the proper completion of surveillance instruments beyond a bureaucratic issue, and to allow an effective epidemiological analysis of TB in the country [31]. Information systems are essential for the epidemiological surveillance of any disease, because through them the information-decision-action process is triggered [32], and the quality of the data is important for the evaluation of services and decision-making in TB control actions [1].

The absence of information about some important variables for tuberculosis monitoring and its non-use in the logistic regression model can compromise the quality of the analysis. We chose to perform the model without considering such variables since excluding the number of entries without this information would considerably reduce the size of our sample and for this reason would be a limitation of our study. We suggest that further studies be done in order to consider these variables.

We also recommend that better attention be given to people at higher risk of abandoning TB treatment in Ananindeua, i.e., individuals with a history of alcoholism and illicit drug use. These cases should be prioritized within the local tuberculosis control program, including priority for the performance of the DOT, along with other priority groups such as homeless people, deprived of freedom and diabetics, as recommended by the Brazilian Ministry of Health [1]. For this, we also recommend the adoption of risk stratification for treatment abandonment and shared care with other services that meet the specific needs of each priority group, such as the stratification proposed by Navarro and collaborators that decreased by fifteen times the chances of abandonment of tuberculosis treatment [2].

## 5. Conclusions

The incidence of TB in Ananindeua showed a similar observation like that observed in the state of Pará and Brazil, although in higher numbers, with 2019 being the one with the highest number of cases and the highest coefficient. Cure outcomes are below the recommended minimum and the treatment abandonment rate is higher than recommended. In this study, alcoholism was almost 2 times more likely to lead the individual to abandon TB treatment and the use of illicit drugs was almost 3 times more likely. Individuals between 20 and 59 years of age were also more likely to abandon treatment almost twice as often. Factors such as ethnicity, and smoking were associated with risk factors for abandonment, but were not risk factors for the abandonment outcome according to the multivariate analysis. Participation in cash transfer program, disease form, and DOT were not statistically associated with dropout cases. It was not possible to estimate the impact of education level and HIV serology as risk factors for treatment abandonment in the proposed statistical model, given the number of reports in which these variables were ignored.

This study was conducted with secondary data provided by the state epidemiological surveillance, with lack of information on important indicators in some of the entries. For this reason, it is essential to strengthen the epidemiological surveillance so that the data from the entries are correctly filled out and minimize possible discrepancies between the information systems and the reality of public health and thus enable more accurate studies.

## Figures and Tables

**Table 1:** Number of new TB cases notified in the municipality of Ananindeua, state of Pará and Brazil from 2017 to 2021.

Year	Ananindeua	Pará	Brazil
2017	318	3920	74,097
2018	380	4099	76,923
2019	498	4823	77,891
2020	417	4293	68,939
2021	399	4133	68,271
Total	2012	21,268	366,121

Source: SINAN/SESPA/MS.

**Table 2:** Closure situation of tuberculosis cases in the municipality of Ananindeua-Pa from 2017 to 2021 (*n* = 2339).

Outcome	2017	2018	2019	2020	2021	Total
Cure	258 (70.1)	309 (68.8)	371 (65.5)	296 (60.9)	135 (28.7)	1369 (58.5)
Abandonment	27 (7.3)	53 (11.8)	49 (8.7)	54 (11.1)	47 (10.0)	230 (9.8)
Death by TB	5 (1.4)	0 (0.0)	4 (0.7)	8 (1.6)	3 (0.6)	20 (0.9)
Death by other reasons	10 (2.7)	12 (2.7)	11 (1.9)	11 (2.3)	7 (1.5)	51 (2.2)
Transferences	28 (7.6)	56 (12.5)	61 (10.8)	30 (6.2)	23 (4.9)	198 (8.5)
Diagnosis change	4 (1.1)	6 (1.3)	7 (1.2)	5 (1.0)	0 (0.0)	22 (0.9)
DR-TB	2 (0.5)	4 (0.9)	4 (0.7)	0 (0.0)	1 (0.2)	11 (0.5)
Schema change	0 (0.0)	2 (0.4)	0 (0.0)	1 (0.2)	2 (0.4)	5 (0.2)
Primary abandonment	0 (0.0)	0 (0.0)	1 (0.2)	0 (0.0)	1 (0.2)	2 (0.1)
Blank	34 (9.2)	7 (1.6)	58 (10.2)	81 (16.7)	251 (53.4)	431 (18.4)
Total	368	449	566	486	470	2339

Data is shown as *n* (%). TB: tuberculosis; DR-TB: drug-resistant tuberculosis.

**Table 3:** Socioeconomic characteristics of the cases with the Treatment abandonment outcome and Tuberculosis cure in Ananindeua from 2017 to 2021.

Indicators	Cases Number (*n* = 1599)	Abandonment (*n* = 230)	Cure (*n* = 1369)	*p*-Value
Sex				
Female	630 (39.4)	76 (33.0)	554 (40.5)	0.0395 ^a^
Male	969 (60.6)	154 (67.0)	815 (59.5)	
Age				
≤19 y.o	179 (11.2)	16 (7.0)	163 (11.9)	0.0062 ^a^
20 to 59 y.o	1223 (76.5)	194 (84.3)	1029 (75.2)	
≥60 y.o	181 (11.3)	17 (7.4)	164 (12.0)	
Ignored	16 (1.0)	3 (1.3)	13 (0.9)	
Ethnicity				
White	172 (10.8)	16 (7.0)	156 (11.4)	<0.0001 ^b^
Black	148 (9.3)	29 (12.6)	119 (8.7)	
Yellow	7 (0.4)	2 (0.9)	5 (0.4)	
Pardo/brown	1229 (76.9)	174 (75.7)	1055 (77.1)	
Indigenous	4 (0.3)	1 (0.4)	3 (0.2)	
Ignored	39 (2.4)	8 (3.5)	31 (2.3)	
Schooling				
Illiterate	21 (1.3)	3 (1.3)	18 (1.3)	<0.0001 ^b^
Primary incomplete	466 (29.1)	95 (41.3)	371 (27.1)	
Elementary School complete	309 (19.3)	44 (19.1)	265 (19.4)	
High School complete	365 (22.8)	26 (11.3)	339 (24.8)	
College degree complete	68 (4.3)	2 (0.9)	66 (4.8)	
Ignored	370 (23.1)	60 (26.1)	310 (22.6)	
Income Transfer Program				
Yes	127 (7.9)	15 (6.5)	112 (8.2)	0.4439 ^a^
No	1384 (86.6)	204 (88.7)	1180 (86.2)	
Ignored	88 (5.5)	11 (4.8)	77 (5.6)	

Data is shown as *n* (%). ^a^: Chi-square test; ^b^: Test G.

**Table 4:** Epidemiological characteristics of the cases with the Treatment abandonment outcome and Tuberculosis cure in Ananindeua from 2017 to 2021.

Indicators	Cases Number (*n* = 1599)	Abandonment (*n* = 230)	Cure (*n* = 1369)	*p*-Value
Type of entry/admission				
New Case	1389 (86.9)	186 (80.9)	1203 (87.9)	<0.0001 ^a^
Recurrence	76 (4.8)	12 (5.2)	64 (4.7)	
Re-entry after abandonment	45 (2.8)	19 (8.3)	26 (1.9)	
Transfer	86 (5.4)	12 (5.2)	74 (5.4)	
Don’t know	3 (0.2)	1 (0.4)	2 (0.1)	
Disease form				
Pulmonary	1427 (89.2)	214 (93.0)	1213 (88.6)	0.0581 ^a^
Extrapulmonary (or mixed)	172 (10.8)	16 (7.0)	156 (11.4)	
DOT				
Yes	229 (14.3)	18 (7.8)	211 (15.4)	0.1735 ^a^
No	1276 (79.8)	142 (61.7)	1134 (82.8)	
Ignored	94 (5.9)	70 (30.4)	24 (1.8)	
Alcoholism				
Yes	178 (11.1)	51 (22.2)	127 (9.3)	<0.0001 ^a^
No	1382 (86.4)	171 (74.3)	1211 (88.5)	
Ignored	39 (2.4)	8 (3.5)	31 (2.3)	
Smoking				
Yes	156 (9.8)	46 (20.0)	110 (8.0)	<0.0001 ^a^
No	1417 (88.6)	178 (77.4)	1239 (90.5)	
Ignored	26 (1.6)	6 (2.6)	20 (1.5)	
Illicit Drugs				
Yes	105 (6.6)	38 (16.5)	67 (4.9)	<0.0001 ^a^
No	1459 (91.2)	184 (80.0)	1275 (93.1)	
Ignored	35 (2.2)	8 (3.5)	27 (2,0)	
HIV				
Positive	145 (9.1)	35 (15.2)	110 (8.0)	<0.0001 ^a^
Negative	697 (43.6)	67 (29.1)	630 (46.0)	
Not performed	757 (47.3)	128 (55.7)	629 (45.9)	

Data is shown as *n* (%). ^a^: Chi-square test; DOT: Directly Observed Treatment; HIV: Human Immunodeficiency Virus.

**Table 5:** Results of the logistic analysis of risk factors for abandoning Tuberculosis treatment in Ananindeua from 2017 to 2021 (*n* = 1345).

Variable	Univariate Analysis			Multivariate Analysis		
	OR	95% CI	*p*-Value	OR	95% CI	*p*-Value
Gender (male vs. female)	1.31	0.94–1.83	0.107			
Age (20 to 59 vs. other ages)	1.57	1.04–2.38	0.033	1.55	1.03–2.32	0.035
Ethnicity (brown vs. other ethnicities)	1.02	0.69–1.51	0.923			
Type of entry/admission (new case vs. other types of entry)	0.49	0.31–0.78	0.002			
Alcoholism (yes vs. no)	2.72	1.68–4.40	<0.001	1.74	1.11–2.72	0.016
Smoking (yes vs. no)	3.33	1.73–6.41	<0.001			
Illicit Drugs (yes vs. no)	4.23	2.35–7.60	<0.001	2.64	1.56–4.46	<0.001
Year (2018 vs. 2017)	0.77	0.53–1.12	0.171			
Year (2019 vs. 2017)	1.13	0.81–1.57	0.482			
Year (2020 vs. 2017)	0.82	0.59–1.15	0.254			
Year (2021 vs. 2017)	1.47	1.03–2.09	0.032	1.45	1.01–2.08	0.043

OR: Odds ratio; CI: Confidence interval.

## Data Availability

All relevant data is presented within the manuscript.
